# Regional Waste Streams as Potential Raw Materials for Immediate Implementation in Cement Production

**DOI:** 10.3390/ma13235456

**Published:** 2020-11-30

**Authors:** Matea Flegar, Marijana Serdar, Diana Londono-Zuluaga, Karen Scrivener

**Affiliations:** 1 Department of Materials, Faculty of Civil Engineering, University of Zagreb, 10000 Zagreb, Croatia; matea.flegar@grad.unizg.hr; 2 Laboratory of Construction Materials, École Polytechnique Fédérale de Lausanne, 1015 Lausanne, Switzerland; diana.londonozuluaga@epfl.ch (D.L.-Z.); karen.scrivener@epfl.ch (K.S.)

**Keywords:** supplementary cementitious materials, industrial by-products, chemical composition, reactivity, compressive strength

## Abstract

There is an urgent need to apply available technologies to reduce the environmental impact of the construction industry. One of the possible solutions that can be implemented immediately is the industrial symbiosis between the waste-producing industries on the one hand and the cement industry, which consumes enormous amounts of raw materials for its production, on the other. In order for the industry to accelerate the use of these available materials and technologies, the potential of these materials must be disclosed. The present study shows a systematic approach to assess the potential of waste materials, by-products, and other raw materials available in the South East Europe that can be used in cement production. Their evaluation included the analysis of their availability, their chemical and physical properties, their chemical reactivity, and their contribution to the mortar’s strength. Based on the results and the analyses carried out, a recommendation for immediate use in the construction sector is given for each of the materials collected.

## 1. Introduction

Year after year, concrete has stood as the most used building material. Its ability to be produced rather cheaply everywhere on earth with good durability and strength properties allows for the production of more than 10 billion tons annually [1]. This massive production comes with an environmental cost, with the cement industry responsible for up to 8% of all CO_2_ emissions generated worldwide [2]. According to the recent report of the United Nations Environmental Program Sustainable Building and Climate Initiative (UNEP-SBCI) [3], the main drivers to reduce these numbers are an increase in energy efficiency and the reduction of the clinker factor. The cement production process has increased its energy efficiency in recent years [4], which suggests that the use of alternative materials for reducing the clinker factor should stand as a key solution. These alternative materials, known as Supplementary Cementitious Materials (SCMs), are proposed as partial cement clinker replacement for the reduction of environmental impact without compromising the mechanical or durability properties of cement composites. Industrial by-products such as fly ash [5], steel slag [6], and silica fume [7] have already been researched and are being used in some types of cement. At the same time, new materials that could serve as partial replacements of cement are being researched intensively, which leads to a broad range of possibilities.

Percentage of cement replacements by industrial waste has been constant for the last couple of decades, roughly remaining on 20% [8]. Research findings suggest there are solutions in going even further than 50% of substitution without compromising the quality or durability of cementitious composites [9,10]. A broad range of characterization [11] and reactivity prediction methods [7,12] for SCMs are already available and well understood. These have been broadly reviewed and accepted by researchers worldwide [13,14,15]. To move to a large-scale production of cements with higher SCM content, research findings should be directly applied in the industry. By forming industrial symbiosis between industries producing waste or by-product with cement plants in close vicinity, we can accelerate the implementation of such materials. For that reason, the scope of this research was to evaluate regionally available industrial waste streams as potential raw materials for construction industry. This research serves as a systematic overview of local waste streams available in sufficient quantities, which can be immediately used as an ecological contribution in the building industry.

## 2. Materials and Methods

### 2.1. Materials

**Figure 1:**
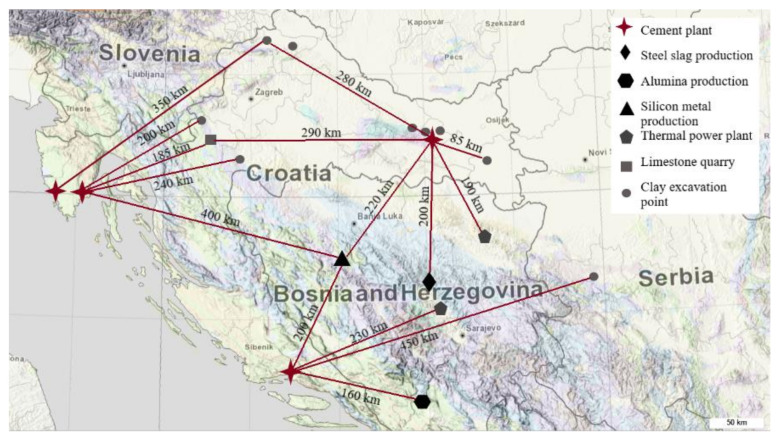
Map of the region [16] with indicated locations of industrial waste materials or excavations sites and their distances from four cement producers in Croatia.

The analysed materials were generated in different industrial processes in the South-East Europe region. Following industries in the region were considered: steel production, thermal power plant, stone quarry, aluminium production, copper production, silicon production and clay excavation for the purpose of ceramic and brick production. Figure 1 indicates a location map of all industries whose waste materials were analysed in this paper. Additionally, four main cement production plants are also indicated on the map.

From these industries, twenty-one different types of possible raw materials generated as waste, were chosen for this research. Industrial residue materials included two slags generated during the steel production process, basic oxygen furnace (SL_BOF) and ladle slag (SL_LF), both from Arcelormittal, Zenica, Bosnia and Herzegovina; one sample of copper production by-product, iron silicate fines (SL_AUR), from Aurubis, Pirdop, Bulgaria; one red mud sample (RM) originating from alumina production Dobro Selo, Bosnia and Herzegovina; two fly ashes—coal combustion products from two thermal power plants in Tuzla and Kakanj, Elektroprivreda, Bosnia and Herzegovina (respectively labeled FA_T and FA_K); and one silica fume densified sample (SF_D), from R-S Silicon, Mrkonjić Grad, Bosnia and Herzegovina product of the silicon alloy production. Natural resources were also a part of this research containing one limestone sample from stone quarry (LS), Zvečaj, Arkada, Croatia, and twelve clay soils, all labeled according to Table 1. Samples of all materials were collected in the period from April to November 2019. The materials were collected from open landfills and were stored in plastic containers. Before the analysis, all collected materials were dried in an aerated oven for 24 ± 2 h at 60 °C to remove moister and milled for 30 s in a disc mill. Clay samples prior to reactivity analysis were additionally calcinated at 800 °C for 1 h [17]. For all the methods presented in this research a sample of ordinary Portland cement, Cem I 42.5 R (OPC) and quartz powder (Q) were used as reference samples to compare particle characterisation and reactivity.

### 2.2. Characterisation Methods

Characterization of raw materials was performed in the Laboratory of Construction Materials at EPFL, Switzerland and in the Laboratory for advanced testing of materials at Department of Materials, Faculty of Civil Engineering, University of Zagreb. The chemical composition of the selected materials was determined by X-ray fluorescence (XRF) following the ISO standard [18] for determination of silicon, aluminium, iron, calcium, magnesium, sodium, potassium, titanium, manganese, phosphorus and sulphur amounts. The density of particles after drying was measured by Le Chatelier flask method according to the ASTM C188-17 standard [19], measuring the change in volume of petroleum when introducing a known amount of powder. The limiting values for evaluating and comparing chemical composition were taken from the European standard for fly ash EN 450-1: 2012 [20]. The particle size distribution (PSD) was determined by Mastersizer 2000 instrument (Malvern Panalytical, Malvern, United Kingdom) with a wet laser diffraction procedure by dispersing the particles in different solvents depending on the material type. The solvents were chosen according to the recommendations from Scrivener et al. [21]. Cement, slags, iron silicate fines and fly ash were dispersed in isopropanol, for clays and red mud dispersing agent was sodium carbonate dissolved in water (pH > 10), for silica fume a 25 wt.% polycarboxylate ether was used, while water was the dispersing agent for limestone and quartz. The morphologies of particles were observed using scanning electron microscopy (SEM) with Tescan Vega III Easyprobe microscope (Tescan, Brno-Kohoutovice, Czech Republic). The micrographs were obtained in high vacuum, under pressure and with high voltage (5 kV).

Thermogravimetric analysis (TGA) was performed with TGA/SDTA 851 apparatus (Mettler Toledo, Columbus, OH, USA). Samples of 50 ± 5 mg of materials were heated from 30 °C to 1000 °C, with a constant heating rate of 10 °C and a nitrogen flow of 30 mL/min. Portlandite (Ca(OH)_2_), calcium carbonate (CaCO_3_) and kaolinite content were calculated based on the mass loss in ranges 300–550 °C, 350–650 °C, and 600–900 °C, respectively. The quantification of these constituents was performed with a tangential calculation of mass loss (${\mathrm{WL}}_{\mathrm{Ca}{\left(\mathrm{OH}\right)}_{2}},{\mathrm{WL}}_{{\mathrm{CaCO}}_{3}},{\mathrm{WL}}_{\mathrm{Kaolinite}}$) multiplied by the molar masses of degrading components ($m_{\mathrm{Ca}{\left(\mathrm{OH}\right)}_{2}},m_{H_{2}O},m_{{\mathrm{CaCO}}_{3}},m_{{\mathrm{CO}}_{2}},m_{\mathrm{Kaolinite}}$) following listed equations [21,22]:

(1) $$\mathrm{Ca}{\left(\mathrm{OH}\right)}_{2,\mathrm{measured}}={\mathrm{WL}}_{\mathrm{Ca}{\left(\mathrm{OH}\right)}_{2}}\times \frac{m_{\mathrm{Ca}{\left(\mathrm{OH}\right)}_{2}}}{m_{H_{2}O}}={\mathrm{WL}}_{\mathrm{Ca}{\left(\mathrm{OH}\right)}_{2}}\times \frac{74}{18}$$

(2) $${\mathrm{CaCO}}_{3,\;\mathrm{measured}}={\mathrm{WL}}_{{\mathrm{CaCO}}_{3}}\times \frac{m_{{\mathrm{CaCO}}_{3}}}{m_{{\mathrm{CO}}_{2}}}={\mathrm{WL}}_{{\mathrm{CaCO}}_{3}}\times \frac{100}{44}\;$$

(3) $${\mathrm{Kaolinite}}_{\;\mathrm{measured}}={\mathrm{WL}}_{\mathrm{Kaolinite}}\times \frac{m_{\mathrm{Kaolinite}}}{2m_{H_{2}O}}={\mathrm{WL}}_{\mathrm{Kaolinite}}\times \frac{256}{36}$$

X-ray diffraction (XRD) was used to quantify the mineralogical composition using the Philips X‘Pert Pro (Malvern Panalytical, Malvern, UK), θ-θ configuration, wavelength CuKα1,α2 with a circular backloading holder and an automatic charging system. Powder pattern were recorded between 5 and 70° 2θ range, with 0.002° 2θ step size and a sampling time per step of 30 s. X’Pert HighScore Plus software (v3.0e, Malvern Panalytical, Malvern, UK) was applied for mineral phase identification and Rietveld refinement. For amorphous quantification high crystalline rutile was used as external standard method.

The reactivity of materials was studied with a calorimetry test developed for the RILEM TC-267 committee [23], called the R3 test. The samples of pastes containing SCMs, sulphate and alkali addition were placed in an isothermal calorimeter at a temperature of 40 °C for 7 days, which resulted in obtaining the total heat release. For each mix, a ratio of Ca(OH)_2_/SCM and CaCO_3_/SCM was 3 and ½, respectively, whereas the alkali solution was made with 3M of K in form of KOH and K_2_SO_4_. All materials and reagents were weighted, mixed and held at 40 ± 2 °C for 24 h prior the experiment. A high shear mixer was used with 1600 ± 50 rpm for 2 min to ensure a homogenous paste, which was immediately cast in glass vial and placed in an isothermal calorimeter. Additionally to the heat release, bound water was calculated by mass change measurement as explained in the mentioned literature [23].

Furthermore, compressive strength test of mortars was used for final comparison. Twenty-one mortar mixes were prepared with a 30% of CEM I 45.2 substitution by collected raw materials. A constant 0.5 water to binder ratio was used for mortar preparation with standardized sand as aggregate. Each mixture was mixed in a 1.5 L batch, following the mixing procedure described in the standard EN 196-1 [24], from which 40 × 40 × 160 mm samples were cast. After casting, samples were held covered in laboratory conditions for 24 h, followed by demolding and underwater curing until testing time. Compressive tests were performed on 2 prisms for each mix after 2, 7 and 28 days of curing age.

## 3. Results

### 3.1. Physicochemical Properties

**Figure 2:**
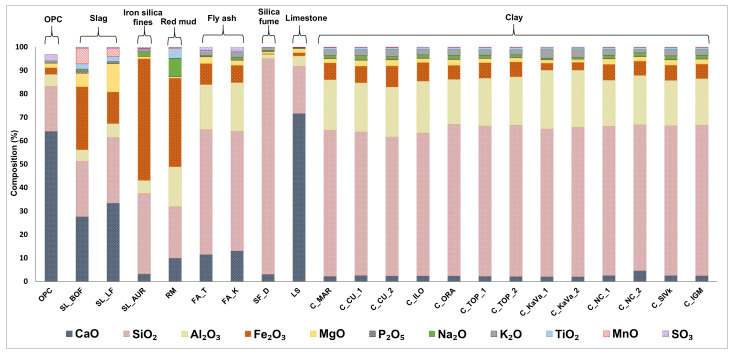
Chemical and physical characteristics of collected raw materials with limitations according to the European standard for fly ash for concrete EN 450:1.

The chemical composition, fineness, crystalline structure, presence of alkalis, etc., are some of the factors influencing the rate and extent of reactions of various mineral admixtures. The chemical composition of all materials is given in Figure 2.

Based on the chemical composition, limiting values used for evaluating fly ash for concrete were expressed according to the European standard for fly ash for concrete EN 450:1 [20], explained in Table 1. These characteristic values include the following limitations: the sum of pozzolanic oxides (SiO_2_, Al_2_O_3_ and Fe_2_O_3_), Na_2_O equivalent, MgO content, SO_3_ content and P_2_O_5_ content.

Most collected materials have the required sum of pozzolanic oxides greater than 70%. Steel slags SL_BOF and SL_LF, and limestone (LS) are the only materials with lower amount of pozzolanic oxides. Iron oxide is the most common oxide in BOF slag, while both slags also contain a certain amount of calcium and magnesium oxides, which could influence the volume stability in pastes. Both iron silicate fines and red mud are rich in iron which is connected to their origin. Limestone, as expected, mostly contains CaO. Silica fume has the highest sum of pozzolans containing mostly silicon dioxide. Regarding the chemical composition and the C618-19 ASTM standard [25] both fly ashes can be defined as class F fly ash having the calcium oxide amount less than 18% as well as the SO_3_ amount less than 5%. All clays have a significant amount of pozzolanic oxides (more than 89%) mostly composed of silicon dioxide and alumina. The mentioned EN standard for fly ash also states requirements for sodium oxide equivalent (Na_2_Oeq), which is obtained from the equation:

(4) $${\mathrm{Na}}_{2}\mathrm{Oeq}={\mathrm{Na}}_{2}O+0.658\times K_{2}O$$

The sodium oxide equivalent for each sample should be less than 5%. All materials satisfy that requirement. There are also the requirements for magnesium, sulphur and phosphorus oxide content being less than 4, 3 and 5% respectively. Only the slags (SL_BOF and SL_LF) do not meet the said limit value for MgO.

**Figure 3:**
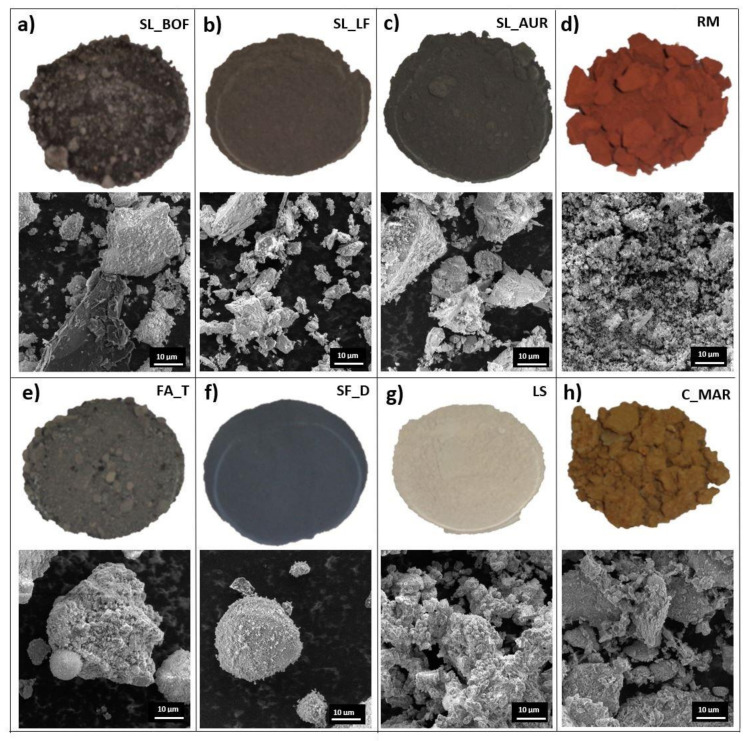
Visual appearance of as-collected materials from different industries (**top image**) and their particle appearance obtained with SEM (**bottom image**) used for morphological comparison: (**a**) BOF slag (SL_BOF), (**b**) ladle slag (SL_LF), (**c**) iron silica fine (SL_AUR), (**d**) red mud (RM), (**e**) fly ash (FA_T), (**f**) silica fume (SF_D), (**g**) limestone (LS), (**h**) clay (C_MAR).

Figure 3 shows images and particle morphology of the samples (only one clay and one fly ash samples are shown as a representative). Images of raw materials prior to treatment indicate the differences in the appearance of the materials, while the SEM images reveal the particle size and morphological variations. SEM of slags shows bigger irregular particles, while red mud shows very fine particles. Fine and spherical particles are visible with fly ash and silica fume, but with tendencies to form conglomerates. Clays show bigger conglomerates that need to be broken by drying and milling. Finally, limestone shows range of particle sizes, all irregular in shape.

**Figure 4:**
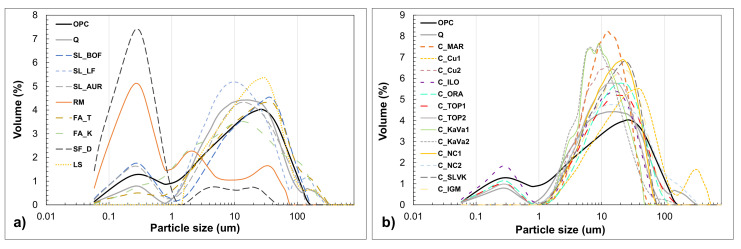
Particle size distribution of selected materials, OPC and quartz obtained by laser diffraction after drying and grinding in a disc mill, (**a**) slags, iron silicate, red mud, fly ashes, silica fume and limestone (**b**) clay samples.

The particle size distribution in volume fraction is shown in the Figure 4a,b. Iron silicate fines and red mud have the highest value of small particles (from 0.1 to 1 µm). The rest of the materials are following similar distribution as the OPC, having the majority of particles around 10 µm of size. Fly ash FA_T, limestone (LF) and slag (BOF) are showing slightly different curves of distribution with a higher middle value. Clay particle distribution is shown in the Figure 4b. Some of the clay samples show a certain number of particles within the size from 0.1 to 1 µm while others do not show the same distribution. There are some differences in the span of particle size, C_CU_1 has a slightly wider distribution while C_MAR, C_KaVa_1 and C_KaVa_2 show higher volume frequency than the others. The differences and similarities between materials and OPC particles can be seen from the median D (v, 0.5) diameter, the tenth percentile D (v, 0.1) and the ninetieth percentile D (v, 0.9). These parameters define which is the exact particle size that divides the distribution into two portions, but also the diameter of particles at which 10% or 90% of particles are smaller.

Table 2 shows density and characteristic values of particle size distribution, while the full particle size distribution is shown in Figure 4a,b. The density of all materials is ranging between 2.08 and 3.69 g/cm^3^, with most of the materials having smaller density than the OPC (3.09 g/cm^3^). The slags SL_BOF and SL_LF have the highest values 3.63 and 3.04 g/cm^3^, respectively. The fly ash Fa_K has a higher density than the Fa_T which shows the lowest result (2.08 g/cm^3^). All clays have a similar density of around 2.5 g/cm^3^.

### 3.2. Phase Composition

**Figure 5:**
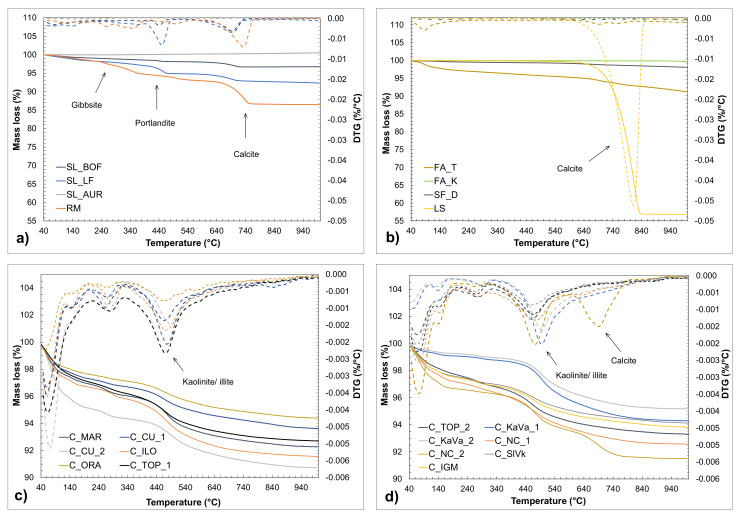
Mass loss and derivative of mass loss (DTG) of raw materials obtained by heating the samples from 40 °C to 1000 °C, with indicated main phases observed: (**a**) slags, iron silicate fines and red mud (**b**) fly ashes, silica fume and limestone, (**c**,**d**) clays.

Figure 5 shows the results acquired by thermogravimetric analysis. The derivative of the mass loss curve emphasizes the crucial periods of mass loss. Considering the different chemical composition of the obtained materials, the derivative curves are significantly different. The initial weight loss at temperatures up to 200 °C, which is present at almost all samples, indicates the moisture content of the material and the weight loss due to the evaporation of free water. Figure 5a shows the mass losses of slags and iron silicate, where two distinguished peaks can be observed, both connected to the slags. The first peak is in the range 300–500 °C, which refers to the dehydroxylation of portlandite (Ca(OH)_2_) and the evaporation of water in that period. The second peak (600–800 °C) is connected to decarbonation of calcium carbonate. Both slags indicate a certain amount of these two minerals, while SL_LF shows a slightly bigger mass loss connected to portlandite content. The slags are collected from an open landfill which could lead to a reaction of water or moisture with the possible reactive phases. Iron silicate fines (SL_AUR) show a slight increase in mass which can be caused by the reaction of sulphur (S_2_^−^), hydrogen sulphide (H_2_S) and oxygen forming sulphates, but also due to the oxidation of iron or manganese [26]. The red mud sample shows two small peaks forming around 250 and 340 °C, which could be assigned to the partial decomposition and transition of mineral gibbsite [27].

The more significant DTG peak appears around 730 °C, which could be attributed to the decarbonation of calcium carbonate (CaCO_3_). The same can be observed in Figure 5b, where the biggest mass drop is connected to the decarbonation of limestone around 800 °C, indicating the CaCO_3_ content. FA_T shows a slight mass loss in the calcite temperature range, different to FA_K and silica fume which show almost no mass changes. Clays, except the peaks of the DTG curve connected to the moisture content, show a significant mass loss in the temperature range between 350° and 670 °C. This mass loss is due to the dehydroxylation process of clay minerals, most likely kaolinite and/or illite [17]. The kaolinite content was approximated and calculated according to the mentioned equation and presented in the Table 3. The results indicate a rather low kaolinite content compared to the literature [9,28].

**Figure 6:**
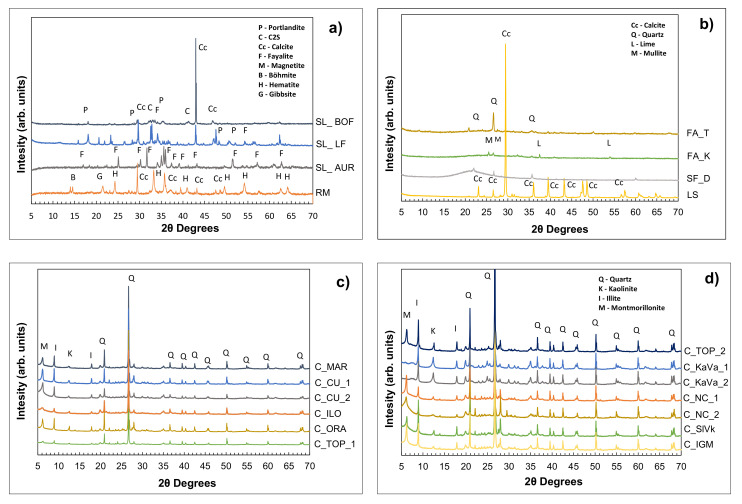
XRD pattern of raw materials: (**a**) slags, iron silicate fines and red mud (**b**) fly ashes, silica fume and limestone, (**c**,**d**) clays with indicated main identified phases.

Figure 6a–d shows the diffractograms of materials obtained from the XRD analysis, which was followed by Rietveld analysis.

The first figure shows the intensity peaks from steel slags, iron silicate and red mud. Main mineral components that can be found in both slags are tricalcium silicate (C_3_S), olivine (C_2_S), portlandite and calcite which give some weak cementitious properties. SL_LF has a higher amount of the mentioned minerals while SL_BOF has a higher amorphous content. Iron silica fines from copper production has a different mineralogical composition, mostly containing Fayalite, Magnetite and Fe-diopside and almost 40% of amorphous phase. Hematite and Gibbsite are highly visible in red mud sample that also contains some amount of calcium carbonate. These non-reactive minerals, together with the low amount of amorphous phase, could negatively influence the strength development of red mud-based concrete [29]. Both fly ashes have a higher amount of amorphous phase, around 90%. FA_T contains some quartz as the first most frequent mineral, while a small amount of free lime can be detected in FA_K. As expected, silica fume, made mostly from silica, has the highest amount of amorphous (>97%), while limestone is mostly containing calcite (87%).

Figure 6c,d shows the XRD diffractograms obtained from all clay samples. The specific peaks indicate the presents of quartz (Q), kaolinite (K), illite (I) and other minerals in smaller quantities. The mineralogical composition implies a higher amount of illite in most clays, a mineral considered to be less reactive than kaolinite. Clays with a higher kaolinite than illite content according to the XRD analysis are C_MAR, C_TOP_2, C_KaVa_1, C_KaVa_2 and C_NC_2.

### 3.3. Chemical Reactivity

**Figure 7:**
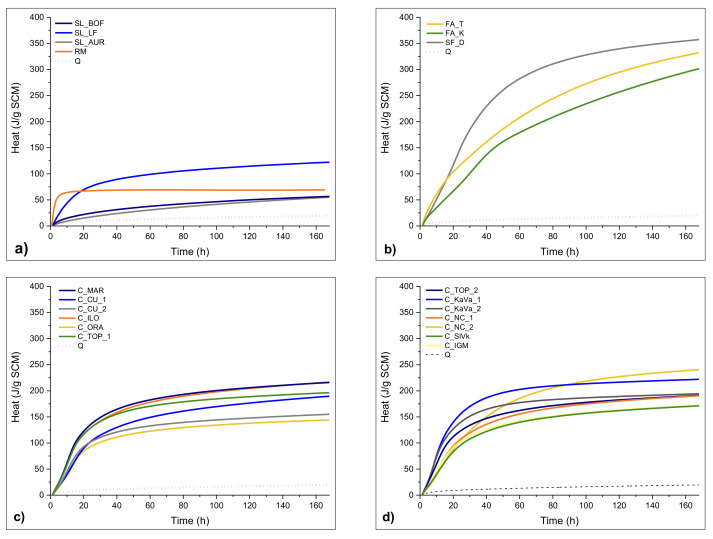
Heat of hydration obtained by R3 test using calorimetry during seven days: (**a**) slags, iron silicate fines and red mud (**b**) silica fume, limestone and fly ashes, (**c**,**d**) clays.

**Figure 8:**
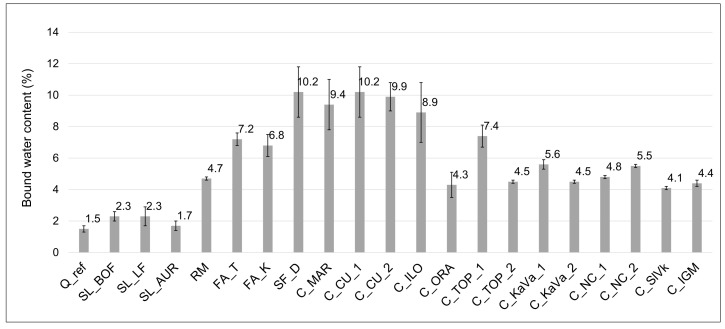
Bound water content for each SCM obtained by the R3 test for reactivity.

Figure 7 and Figure 8 show the results of reactivity tests—the R3 test [30], by isothermal calorimetry and bound water, respectively. The test results of different raw materials are presented in comparison with the inert quartz sample (Q). 

In Figure 7a, the slag LF shows best results amongst others, with higher amount of heat released through the 7 days but also a higher initial reaction in the first 20 h. Still, this is a rather low amount of heat compared to the nonreactive reference. The other slags SL_AUR and SL_BOF show slightly higher overall results than the inert quartz, showing linear heat release from the beginning. The red mud sample has the highest initial heat release, most likely due to the alkali reaction at early stages, after which the heat stays at a constant value. The bound water test results, presented in Figure 8, differ slightly from the isothermal calorimetry test results in these cases, showing the same values for BOF and LF slag, and somewhat higher values for red mud. Silica fume again shows the highest end value but interestingly has a slower initial reaction than the FA_T sample. Both fly ashes show similar heat release after 7 days and a similar continuity during the experiment, but FA_T holds a higher end value. The results for the FA_K imply a slower initial and overall reaction. All results shown in the Figure 7b hold a significantly higher reactivity than the reference quartz. Before reactivity tests and the grinding and drying, clays were calcined on 800 °C for 1 h to activate their reactivity. All clays show a similar pattern of heat release, a slightly faster release in the first 15 h of reaction followed by a slower reaction after 24 h. From the seven-day results, the highest reactivity values show samples C_NC_2, C_KaVa_1, C_MAR and C_ILO, with more than 200 J/g of SCM. C_ORA, indicate the least reactivity with a heat release after seven days of only 142.6 J/g. Interestingly, the results from the bound water test presented in Figure 8 do not completely comply with the heat release test, showing higher bound water content for clays with a smaller heat of hydration. When comparing the TGA results, the clays show some amount of free water, even though they were previously dried. This could indicate a free water content that affects the bound water test results. The calculation of bound water should be then corrected with the free water contained in the SCMs [30].

**Figure 9:**
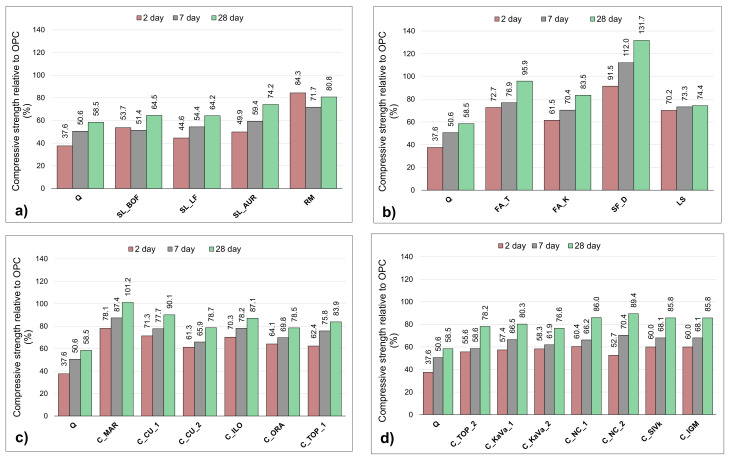
Compressive strength results of mortars with a 30% SCMs substitution relative to compressive strength of OPC sample: (**a**) slags, iron silicate fines and red mud (**b**) silica fume, limestone and fly ashes, (**c**,**d**) clays.

The strength test of mortars prepared with a 30% cement substitution shown in the Figure 9a–c corresponds to the R3 test results.

Silica fume has a slightly lower initial strength but then exceeds the OPC sample results. Almost reaching the OPC strength after 28 days, FA_T shows the third-best results overall. Initially with a higher strength than the FA_K, both fly ashes show almost the same increase. Mortar sample containing limestone shows surprisingly good results, performing even better than the slags. When looking at all of the 28-day strengths results clay sample C_Mar performs the second best, exceeding the OPC in later ages. The initial strength of this sample is rather lower, as for all clays, reaching around 80% of the OPC 2-day strength. Confirming the bound water test but not matching to the heat release the C_CU_1 sample shows rather high results, almost the same strength results as the C_NC_2 sample. Except the C_MAR sample, none of the clays reach the OPC strength, but all of them follow the same trend of higher strength gain rate. The initial strengths are low, 50 to 80% of that form the OPC, slightly catching up to the 28-day OPC strength with a linear increase.

## 4. Discussion and Conclusions

When characterizing the chemical composition, pozzoloanicity of SCMs is generally relying on a high silica and alumina content. All tested materials except steel slags and limestone meet the limiting requirements laid down in EN 450:1 standard for fly ash. This indicates their possibilities to be used in standardized cement. Obviously, chemical composition cannot be looked as a solely indicator of pozzolanic properties. Amorphous content has proven to be a more reliable predictor of long-term strength and activity of natural pozzolans [31]. Clays show a high sum of pozzolanic oxides (around 90%) but have relatively little content of amorphous phases (no more than 15%). Fly ash samples and silica fume, on the other hand, show the highest amorphous content. Particle size of materials gives an indication of their role in the hydration process when used as partial cement replacements. Smaller particles add to the so-called “filler effect”, a physical effect of providing additional surface for hydrates to participate [21]. Red mud and silica fume are such materials, where an increased early compressive strength (2 days) can be seen. Phase composition, studied with the TGA analysis combined with XRD, indicated portlandite in both slags (LF and BOF) but also calcite, which was to some extend expected, due to long open-air landfilling of these materials. Some clays on the other hand, seem to have significant content of quartz particles. Furthermore, they have smaller content of kaolin, which identifies them as a low grade kaolinitic clays.

Reactivity of samples was studied through the R3 test (isothermal calorimetry and bound water test), and the compressive strength of mortar. Silica fume, FA_T fly ash and clay C_MAR are the most reactive samples, reaching and/or overachieving the strength of the OPC sample after 28 days. All three samples show high heat release and higher bound water content, compared to all other materials. Slags BOF and LF are showing results similar to the inert quartz sample which suggests they do not possess pozzolanic properties. Other materials seem to show some reactivity, clays getting from 75 to 90% of the OPC 28 days strength, but with much smaller early age strengths (around 60%). Red mud and silica fume are the only samples showing higher initial reactivity, compared to OPC.

This research reveals there is a possibility of full-scale utilization of the available resources from the region as supplementary cementitious materials. The most promising materials for immediate utilisation in the industry are fly ash, silica fume, and certain clays (C_Mar). Certain types of clays (C_NC1, for example) have a significant content of quartz, and the following step could be separation of their particles, to obtain materials with a higher content of reactive kaolin [32]. Considering the small particle size and initial higher reactivity, red mud could be potentially used with other by-products to achieve synergy [7]. Due to the high alkalinity a combination of red mud with materials such as class F fly ash from this research could give good results when used in geopolymers [29]. Finally, the least reactive materials such as slags, if ground finely could serve as inert filler, contributing to the strength development by providing additional space for nucleation growth, or could be used to improve fire or wear resistance of concrete. Considering the coarse fraction and the hardness of these materials in the raw state, usage of local slags without pre-treatment could be even more economically justified when applied as concrete aggregate [33]. Even the less reactive fly ash (Fa_K) could potentially serve as fine fraction aggregate, as it has already been proven on bottom ash samples from local thermal plants [12].

Presented research showed that the south-east Europe region has substantial reserves of waste streams from various industries that are either produced yearly or stacked on landfills causing ecological and economic burden to the production industries. Even though there are predictions of lowering the production amounts of materials such as coal combustion fly ash and slags, due to the EU regulations, countries such as Bosnia and Herzegovina do not fall under these restrictions and will continue producing such materials years. Secondly, large amounts of these and other waste materials are already produced and stored in hundreds of thousand tons that can be utilized immediately. All materials collected during this research have a possibility of transfer within 500 km to the main cement plants in Croatia, which further enhances the ecological aspect of this research.

## Figures and Tables

**Table 1:** Chemical and physical characteristics of collected raw materials with limitations according to EN 450:1 [20].

Material	∑ Primary Oxides ≥ 70% wt.	Na_2_O_eq_ ≤ 5% wt.	MgO ≤ 4% wt.	SO_3_ ≤ 3% wt.	P_2_O_5_ ≤ 5% wt.
SL_BOF	55.51	0.51	5.68	0.46	1.37
SL_LF	47.45	0.31	12.00	0.44	0.72
SL_AUR	91.74	2.42	0.94	0.53	0.26
RM	76.77	7.35	0.61	0.24	0.47
FA_T	81.44	1.25	2.78	1.48	0.36
FA_K	79.10	2.20	2.15	1.72	0.54
SF_D	94.15	0.94	0.77	0.27	0.36
LS	25.96	<0.010	1.69	0.08	0.42
C_MAR	91.02	2.70	1.78	0.07	0.36
C_CU_1	89.38	3.26	2.42	0.07	0.44
C_CU_2	89.53	3.09	2.57	0.07	0.40
C_ILO	90.99	2.76	1.75	0.08	0.36
C_ORA	89.78	3.18	2.41	0.07	0.40
C_TOP_1	91.11	2.72	1.78	0.09	0.36
C_TOP_2	91.53	2.47	1.74	0.10	0.34
C_KaVa_1	91.12	3.08	1.38	0.07	0.28
C_KaVa_2	91.47	2.97	1.28	0.08	0.28
C_NC_1	90.03	2.92	2.34	0.12	0.35
C_NC_2	89.37	2.29	1.88	0.08	0.35
C_SlVk	89.70	3.18	2.25	0.08	0.43
C_IGM	90.29	2.97	2.11	0.07	0.39

**Table 2:** Density and characteristic particle sizes of collected raw materials.

Material	Density	Particle Size (µm)		
	(g/cm^3)^	D (v, 0.1)	D (v, 0.5)	D (v, 0.9)
OPC	3.09	0.32	9.95	50.8
SL_BOF	3.63	0.2	13.1	57.2
SL_LF	3.04	2.6	10.3	47.2
SL_AUR	2.4	0.2	9.5	46
RM	2.88	0.1	0.4	10.1
FA_T	2.08	1.5	15.2	73
FA_K	2.47	0.7	8.6	68
SF_D	2.13	0.1	0.3	3.5
LS	2.7	3.4	18	63.8
C_MAR	2.26	4	10.7	24.8
C_CU_1	2.59	5	23.8	104.8
C_CU_2	2.43	3.4	10.8	31.3
C_ILO	2.29	0.2	8.9	29.8
C_ORA	2.56	0.4	13	40.6
C_TOP_1	2.52	0.5	12.2	43.5
C_TOP_2	2.5	1.3	10	30.1
C_KaVa_1	2.57	3.2	8.6	24.1
C_KaVa_2	2.57	3	8.2	24.5
C_NC_1	2.12	3.8	13.4	34.7
C_NC_2	2.41	3.5	15.4	72.7
C_SlVk	2.5	3.8	14.1	36.7
C_IGM	2.57	4.1	15.4	44

**Table 3:** Mass loss and mineral content calculation obtained by TGA for different clays.

Material	Mass Loss 350–670 °C (%)	Kaolinite Content (%)
C_Cu1	1.63	11.6
C_Cu2	1.52	10.8
C_ORA	1.31	9.7
C_IlO	2.68	19.1
C_Mar	2.47	17.6
C_TOP 1	1.90	13.5
C_TOP2	2.87	20.4
C_NC_1	2.93	20.8
C_NC_2	2.72	19.3
C_KaVa_1	3.26	23.2
C_KaVa_2	2.85	20.3
C_SLVK	2.11	15.0
C_IGM	2.26	16.1

## References

[1] Meyer C. (2009). The greening of the concrete industry. Cem. Concr. Compos..

[2] World Business Council for Sustainable Development (2016). Cement Industry Energy and CO_2_ Performance: Getting the Numbers Right (GNR). Cem. Sustain. Initiat. Cem..

[3] Scrivener K.L., John V.M., Gartner E. (2018). Eco-efficient cements: Potential economically viable solutions for a low-CO2 cement-based materials industry. Cem. Concr. Res..

[4] Schneider M., Romer M., Tschudin M., Bolio H. (2011). Sustainable cement production—Present and future. Cem. Concr. Res..

[5] Celik K., Meral C., Mancio M., Mehta P.K., Monteiro P.J.M. (2014). A comparative study of self-consolidating concretes incorporating high-volume natural pozzolan or high-volume fly ash. Constr. Build. Mater..

[6] Manso J.M., Losáñez M., Polanco J.A., González J.J. (2005). Ladle Furnace Slag in Construction. J. Mater. Civ. Eng..

[7] Ćećez M., Šahinagić-Isović M. (2019). Mortars with addition of local industrial by-products. Građevinar.

[8] Habert G., Billard C., Rossi P., Chen C., Roussel N. (2010). Cement production technology improvement compared to factor 4 objectives. Cem. Concr. Res..

[9] Avet F., Scrivener K. (2018). Investigation of the calcined kaolinite content on the hydration of Limestone Calcined Clay Cement (LC3). Cem. Concr. Res..

[10] Blotevogel S., Ehrenberg A., Steger L., Doussang L., Kaknics J., Patapy C., Cyr M. (2020). Ability of the R3 test to evaluate differences in early age reactivity of 16 industrial ground granulated blast furnace slags (GGBS). Cem. Concr. Res..

[11] Lothenbach B., Scrivener K., Hooton R. (2011). Supplementary cementitious materials. Cem. Concr. Res..

[12] Janković K., Bojović D., Lončar L., Stojanović M., Antić L. (2018). Possibility of using bottom ash in precast concrete products. J. Croat. Assoc. Civ. Eng..

[13] Snellings R. (2016). Assessing, Understanding and Unlocking Supplementary Cementitious Materials. RILEM Tech. Lett..

[14] Juenger M.C.G., Siddique R. (2015). Recent advances in understanding the role of supplementary cementitious materials in concrete. Cem. Concr. Res..

[15] Snellings R., Mertens G., Elsen J. (2012). Supplementary Cementitious Materials. Rev. Miner. Geochem..

[16] The Geological Survey Organizations of Europe, E-Government Development Index (EGDI). http://www.europe-geology.eu/onshore-geology/geological-map/igme5000/.

[17] Fernandez R., Martirena F., Scrivener K.L. (2011). The origin of the pozzolanic activity of calcined clay minerals: A comparison between kaolinite, illite and montmorillonite. Cem. Concr. Res..

[18] The International Organization for Standardization (2018). ISO 13605:2018 Solid mineral fuels—Major and Minor Elements in Coal Ash and Coke Ash—Wavelength Dispersive X-Ray Fluorescence Spectrometric Method.

[19] ASTM International (2017). ASTM C188-17, Standard Test Method for Density of Hydraulic Cement.

[20] European Standard (2012). EN 450-1:2012 Fly Ash for concrete—Part 1: Definition, Specifications and Conformity Criteria.

[21] Scrivener K., Snellings R., Lothenbach B. (2016). A Practical Guide to Microstructural Analysis of Cementitious Materials.

[22] Avet F., Scrivener K. (2020). Simple and Reliable Quantification of Kaolinite in Clay Using an Oven and a Balance. Calcined Clays for Sustainable Concrete, RILEM Bookseries.

[23] Li X., Snellings R., Antoni M., Alderete N.M., Ben Haha M., Bishnoi S., Cizer Ö., Cyr M., de Weerdt K., Dhandapani Y. (2018). Reactivity tests for supplementary cementitious materials: RILEM TC 267-TRM phase 1. Mater. Struct. Constr..

[24] European Committee for Standardization (CEN) (2016). EN 196-1:2016 Methods of Testing Cement—Part 1: Determination of Strength.

[25] ASTM International (2010). C618—19, Standard Specification for Coal Fly Ash and Raw or Calcined Natural Pozzolan for Use.

[26] Montes-Morán M.A., Concheso A., Canals-Batlle C., Aguirre N.V., Ania M.C.O., Martín M.J., Masaguer V. (2012). Linz-Donawitz Steel Slag for the Removal of Hydrogen Sulfide at Room Temperature. Environ. Sci. Technol..

[27] Atasoy A. (2005). An investigation on characterization and thermal analysis of the Auginish red mud. J. Therm. Anal. Calorim..

[28] Taylor-Lange S.C., Lamon E.L., Riding K.A., Juenger M.C. (2015). Calcined kaolinite–bentonite clay blends as supplementary cementitious materials. Appl. Clay Sci..

[29] Koshy N., Dondrob K., Hu L., Wen Q., Meegoda J.N. (2019). Mechanical Properties of Geopolymers Synthesized from Fly Ash and Red Mud under Ambient Conditions. Crystals.

[30] ASTM International (2020). C1897−20, Standard Test Methods for Measuring the Reactivity of Supplementary Cementitious Materials by Isothermal Calorimetry and Bound Water.

[31] Walker R., Pavía S. (2010). Physical properties and reactivity of pozzolans, and their influence on the properties of lime–pozzolan pastes. Mater. Struct..

[32] Zunino F., Scrivener K. (2020). Increasing the kaolinite content of raw clays using particle classification techniques for use as supplementary cementitious materials. Constr. Build. Mater..

[33] Netinger I., Rukavina M.J., Bjegović D. (2010). Possibility of using domestic slag as concrete aggregate. Gradjevinar.

